# Hybrid optical fiber for light-induced superconductivity

**DOI:** 10.1038/s41598-020-64970-w

**Published:** 2020-05-18

**Authors:** Evgeny Sedov, Irina Sedova, Sergey Arakelian, Giuseppe Eramo, Alexey Kavokin

**Affiliations:** 1 Westlake University, School of Science, 18 Shilongshan Road, Hangzhou, 310024 Zhejiang Province China; 2 Westlake Institute for Advanced Study, Institute of Natural Sciences, 18 Shilongshan Road, Hangzhou, 310024 Zhejiang Province China; 3 Vladimir State University named after A. G. and N. G. Stoletovs, Department of Physics and Applied Mathematics, Gorky str. 87, 600000 Vladimir, Russia; 4 Mediterranean Institute of Fundamental Physics, 31, Appia Nuova, Frattocchi, Rome, 00031 Italy; 5 St. Petersburg State University, Spin Optics Laboratory, Ul’anovskaya 1, Peterhof, St. Petersburg, 198504 Russia

**Keywords:** Bose-Einstein condensates, Semiconductors, Superconducting properties and materials, Surfaces, interfaces and thin films, Polaritons

## Abstract

We exploit the recent proposals for the light-induced superconductivity mediated by a Bose-Einstein condensate of exciton-polaritons to design a superconducting fiber that would enable long-distance transport of a supercurrent at elevated temperatures. The proposed fiber consists of a conventional core made of a silica glass with the first cladding layer formed by a material sustaining dipole-polarised excitons with a binding energy exceeding 25 meV. To be specific, we consider a perovskite cladding layer of 20 nm width. The second cladding layer is made of a conventional superconductor such as aluminium. The fiber is covered by a conventional coating buffer and by a plastic outer jacket. We argue that the critical temperature for a superconducting phase transition in the second cladding layer may be strongly enhanced due to the coupling of the superconductor to a bosonic condensate of exciton-polaritons optically induced by the evanescent part of the guiding mode confined in the core. The guided light mode would penetrate to the first cladding layer and provide the strong exciton-photon coupling regime. We run simulations that confirm the validity of the proposed concept. The fabrication of superconducting fibers where a high-temperature superconductivity could be controlled by light would enable passing superconducting currents over extremely long distances.

## Introduction

The theoretical concept of conventional superconductivity introduced by Bardeen, Cooper and Schrieffer (BCS)^1^ relies on the pairing of electrons in a Fermi sea due to the exchange by quanta of crystal lattice vibration: acoustic phonons. Since 1970s, multiple attempts were made to replace phonons by a more efficient binding agent that would strengthen electron-electron attraction and enable superconductivity at higher temperatures. Excitons have been put forward by Allender, Bray and Bardeen^2^ and Ginzburg^3^ as promising candidates for playing the role of such a binding agent in hybrid metal-semiconductor structures. However, despite of significant efforts to fabricate structures where the exciton-mediated superconductivity would be observable, no experimental evidence for this mechanism was reported till now, to the best of our knowledge. The interest to exciton-mediated superconductivity was renewed after the discovery of the Bose-Einstein condensation of light-matter bosonic quasiparticles, exciton-polaritons, in semiconductor microcavities, initially at the liquid Helium temperature^4^ and then at the room temperature^5^. Exciton-polaritons formed by strongly coupled elementary crystal excitations (excitons) and cavity photons^6^ accumulate by tens thousands in a single quantum state thus giving rise to the polariton-lasing^5^. In a series of theoretical works^7–12^, it was shown that the condensates of exciton polaritons may interact with free electrons much stronger than individual virtual excitons considered in the early works^2,3^. This paves the way to the realisation of exciton-mediated superconductivity in hybrid multilayer structures where a free electron gas would be placed in the vicinity of a bosonic condensate of exciton-polaritons. The observation of Bose-Einstein condensation of exciton-polaritons at the room temperature^5,6^ encouraged efforts for the observation of superconductivity mediated by exciton-polaritons at elevated temperatures. It has been argued^8^ that a stationary dipole polarisation of excitons in the condensates might help maximising the strength of exciton-electron coupling, which is why strongly coupled structures containing spatially indirect excitons might be suitable for the realisation of exciton-mediated superconductivity. A variety of materials potentially promising from this point of view has been considered, including the two-dimensional monolayers of transition metal dichalcogenides^10,11^ and perovskite nanoplatlets^13^. In ref. ^14^ it has been argued that the interplay between conventional BCS superconductivity and the exciton-mediated superconductivity may result in the resonant enhancement of the critical temperature for the superconducting phase transition *T*_c_, thus, potentially, paving the way to room temperature superconductivity that would be fully controlled by light that is used to pump exciton-polariton condensates. The recent experimental studies^15,16^ revealed features of the light-induced superconductivity. It was argued that the phase transition has been triggered by an optically pumped vibron mode that represents a similar mechanism to the exciton-induced superconductivity. Still, no stationary increase of *T*_c_ mediated by laser illumination has been reported so far, to our knowledge.

**Figure 1:**
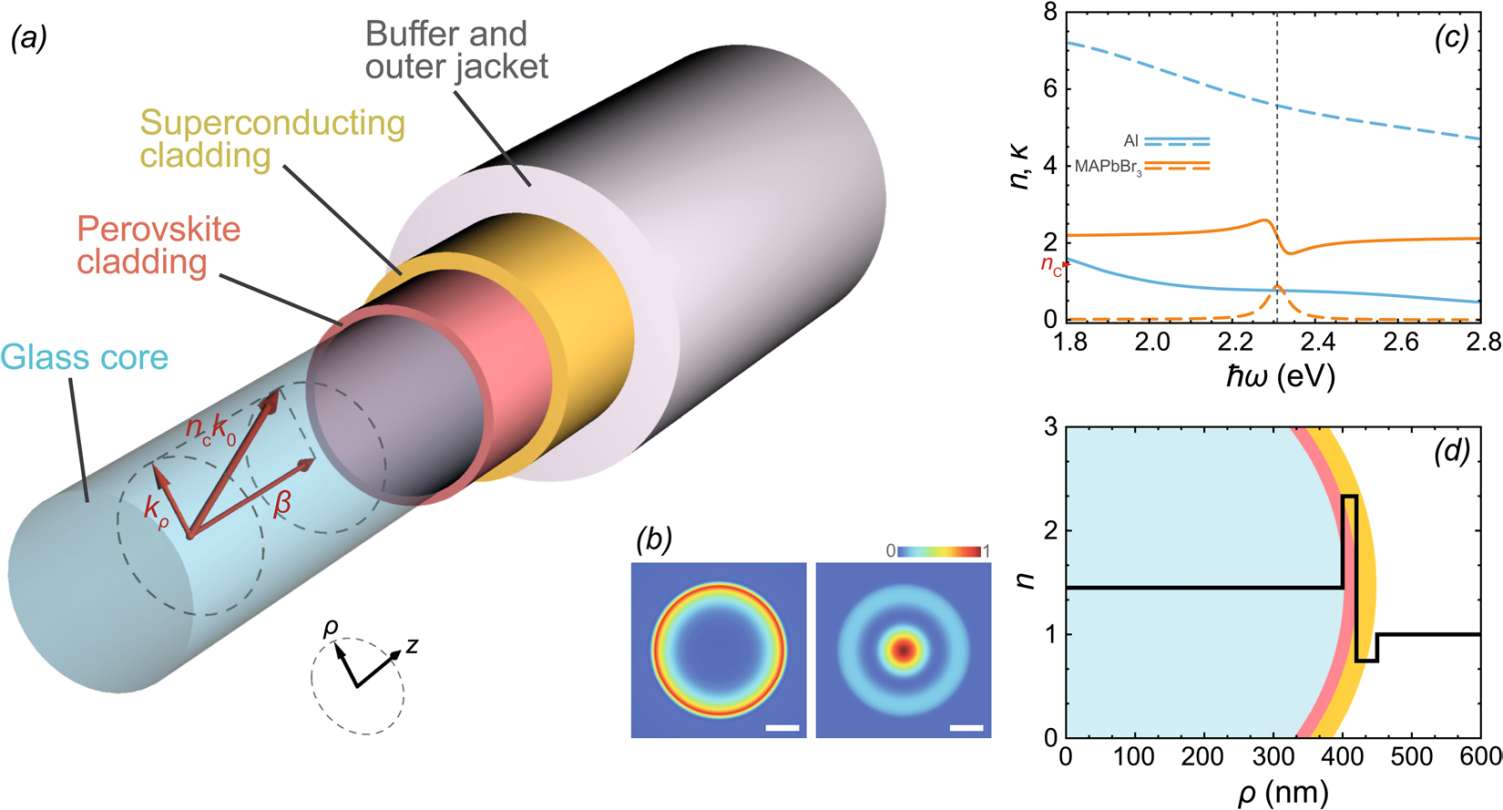
(**a**) The schematic of a proposed design. The bosonic condensate of dipole polarised exciton-polaritons is formed by the optical pumping through the guided optical modes of the fiber. The strong exciton-photon coupling regime is achieved due to the overlap of the photon mode localized in the core with the exciton state located in the first cladding layer (perovskite). The proximity of the perovskite layer to the second cladding layer (superconductor) ensures the efficient coupling of the dipole-polarised condensate of polaritons with the electron Fermi sea in the superconductor. This coupling leads to a significant increase of the critical temperature of the superconducting phase transition. The core and cladding layers are protected by plastic buffer and jacket. The red arrows indicate the decomposition of a guided mode wave vector on the transversal component **k**_*ρ*_ and the propagation constant *β* along the main axis of the fiber. (**b**) The characteristic intensity distribution of the ground plasmonic mode (left) and the ground guided polariton mode (right). The width of the white scale bar corresponds to 200 nm. (**c**) Dispersions of the refractive indices *n* (solid curves) and extinction coefficients *κ* (dashed curves) of the materials used in the proposed hybrid optical fiber. Data for aluminium (cyan) was taken from ref. ^25^. *n* and *κ* of MAPbBr_3_ in the vicinity of the absorption resonance were calculated from Eq. (1) as $n-i\kappa =\sqrt{\varepsilon }$^17^. The red marker indicates the refractive index of the core. The vertical dashed line indicates the perovskite exciton resonance at $\hslash {\omega }_{{\rm{t}}}=2.303$ eV. (**d**) Refractive index profile of the hybrid waveguide in the radial direction. Schematic of the hybrid waveguide cross-section without the outer jacket is put under the curve as a guide for the eye. The figure (a) and all figures in the manuscript were created using Wolfram Mathematica version 11.3, https://www.wolfram.com/mathematicahttps://www.wolfram.com/mathematica.

In this paper, we propose a design of the structure that would enable light-mediated superconductivity triggered by a bosonic condensate of exciton-polaritons in a conventional optical fiber^17^ containing two cladding layers: one made of a material sustaining dipole-polarised excitons that are able to strongly couple to the guided optical mode, and the second one made of a conventional superconductor (see the schematic in Fig. 1(a)). To be specific, we consider a first cladding layer made of a perovskite material where polariton lasing was recently demonstrated in micro- and nanowires^18,19^, and a conventional superconductor aluminium^20^. We note that the exciton binding energy in the considered perovskite material exceeds 25 meV which makes it suitable for the room temperature operation^18^. The critical temperature for the superconducting phase transition in aluminium is about 2 K in the dark, but it may be strongly enhanced by the coupling to the polariton condensate. From the theoretical point of view, the novelty of the considered design is in the replacement of a stationary polariton condensate considered in the previous studies^7–10^ with a moving condensate that may be treated as a coherent quantum liquid^21^. From the practical point of view, the realisation of optical fibers with superconducting cladding layers would pave the way to the long-range transport of supercurrents. The coupling of a superconductor with a condensate of exciton-polaritons that is pumped by a laser light passing through the core would constitute a tool for the enhancement of *T*_c_ as well as the switching mechanism that enables the optical control of superconductivity. It is important to note that while the absorption of light in a superconducting cladder is inevitable, in principle, it can be minimized by proper designing the fiber and compensated due to the pumping of polaritons through one of the higher frequency guided light modes of the fiber. Below we present estimations of the characteristics of the superconducting hybrid fiber and provide specific recommendations for its design.

## Results and Discussion

The proposed hybrid optical fiber is schematically shown in Fig. 1(a). The cylindrical core is assumed being made of a conventional silica glass. For further estimations, we take the refractive index of the core as *n*_C_ = 1.45. The first cladding layer intended to be a holder of excitons is a perovskite layer. Among a variety of perovskites, we give a preference to methylammonium lead tribromide MAPbBr_3_ (MA = CH_3_NH_3_). The strong coupling of excitons with light in the waveguide geometry has been recently reported for the structures where MAPbBr_3_ was used as a core nanowire^18,19^. The peculiar optical and dielectric properties of MAPbBr_3_ in the spectral range from visible to near-ultraviolet have been extensively studied both experimentally and theoretically for last several years^13,19,22^ in the context of developing solar cell circuits and photonic devices. In the photon energy range following immediately above the band gap starting around 2.24 eV and extending to about 4.5 eV, the MAPbBr_3_ film is characterized by three absorption peaks at energies of about 2.3, 3.5 and 4.5 eV^23,24^. Authors of ref. ^23^ show that this peculiarity of the observed spectrum can be described by a dielectric function characterized by four resonances within the indicated energy region. The lowest energy resonance situated near the band gap (at about 2.3 eV) is split from the next one (situated at about 3.5 eV) by more than 1 eV, which significantly exceeds the characteristic interaction energies in our system. This allows one to use the coupled oscillator model of the perovskite dielectric function in the simulations^18^:

1 $${\varepsilon }_{{\rm{P}}}(\omega )={\varepsilon }_{{\rm{b}}}\left(1,+,\frac{{\omega }_{{\rm{l}}}^{2}-{\omega }_{{\rm{t}}}^{2}}{{\omega }_{{\rm{t}}}^{2}-{\omega }^{2}-{\rm{i}}\omega \Gamma }\right),$$

where *ε*_b_ is the background dielectric constant, *ω*_l,t_ are the longitudinal and transverse exciton frequencies, Γ is the non-radiative exciton damping. Values of the parameters for the best fit of *ε*_P_(*ω*) are following^18^: *ε*_b_ = 4.7, $\hslash {\omega }_{{\rm{l}}}=2.328$ eV, $\hslash {\omega }_{{\rm{t}}}=2.303$ eV and $\hslash \Gamma =59$ meV. The refractive index and the extinction coefficient of MAPbBr_3_ found as ${n}_{{{\rm{MAPbBr}}}_{3}}-{\rm{i}}{\kappa }_{{{\rm{MAPbBr}}}_{3}}=\sqrt{{\varepsilon }_{{{\rm{MAPbBr}}}_{3}}}$ are shown in Fig. 1(b) in the vicinity of the exciton resonance at $\hslash {\omega }_{{\rm{t}}}$. We take the thickness of the MAPbBr_3_ cladding layer as 20 nm.

When choosing the second (superconducting) cladding layer, we follow the original paper on the polariton-mediated superconductivity^14^ and consider for this role a thin film of aluminium^20^. Aluminium possesses an advantage of high reflectivity properties, which contributes to reducing losses of the optical modes and strong coupling of the latter to excitons in the perovskite cladding layer. In the simulations, we consider an aluminium film of thickness of 30 nm. The spectral dependences of the refractive index and the extinction coefficient of aluminium taken from ref. ^25^ are shown in Fig. 1(b). The refractive index profile of the hybrid waveguide in the radial direction is schematically shown in Fig. 1(c). The cladding layers can be separated from one another by a spacer of widths of the order of 1 nm with a refractive index matching that of the core.

To reach the effective coupling of the perovskite exciton with a light mode in the waveguide, we should bring one of the optical guided modes into the resonance with the exciton energy. This imposes restrictions on the wave number *β* characterizing the propagation of the guided mode along the main axis of the waveguide, see Fig. 1. For the bare waveguide representing a glass core in air, the condition for *β* in general form is given by *k*_0_ < *β* < *n*_C_*k*_0_ with *k*_0_ = *ω*_0_/*c* and *ω*_0_ being the wave number and the angular frequency of light in vacuum. A guided mode characterized by a frequency of *ω* = *ω*_1_ should possess the wave number *β* belonging to the range of (11.7 *μ*m^−1^, 16.9 *μ*m^−1^). Our simulations show that additional cladding layers shift this range by at least 3.3 *μ*m^−1^ to larger values. In room temperature long-range coherent exciton polariton condensate flow in lead halide perovskites^26^ the propagation of superfluid polariton flows in perovskite microcavity over 60 micrometer distance is reported. We expect that in the proposed fiber geometry, the non-amplified propagation length may be much larger due to the large wave-vectors of a guided mode and a better optical confinement as compared to a planar microcavity.

For our hybrid optical fiber, we propose to take a glass core of a reasonably small diameter being of the order of the guided mode wavelength, ${d}_{{\rm{C}}}\gtrsim \lambda$. In such a waveguide, the higher energy guided modes are split from the ground mode by hundreds of millielectron volts. It allows one to neglect the effect of the higher modes on the coupling of the fundamental mode to the perovskite excitons. We note that higher energy guided modes are crucial for the long distance transmission of polariton superfluids and light-induced superconducting currents. They serve to feed the polariton condensate and compensate for inevitable losses. The amplification of the polariton mode may be done using the schemes used in optical fiber amplifiers based e.g. on the electronic transitions in Er atoms^27–29^.

**Figure 2:**
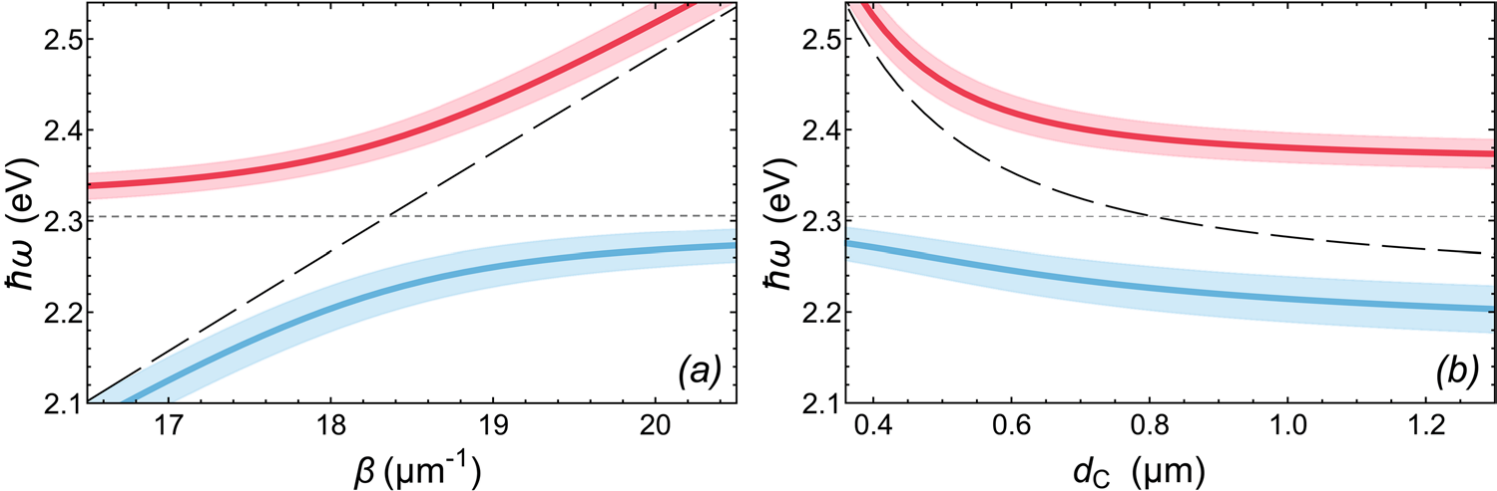
(**a**) The variation of the energy of exciton-polariton modes in the hybrid optical fiber with the increase of the propagation constant *β* and (**b**) the dependence of the energy of exciton-polariton modes in the hybrid optical fiber on its core diameter *d*_C_ calculated using the transfer matrix method described in Methods. The core diameter for (**a**) is taken as *d*_C_ = 0.8 *μ*m. The propagation constant for (**b**) is taken as *β* = 18.4 *μ*m^−1^. The pale color shadows framing the curves indicate the radiative decay of the modes. The widths of the shadows correspond to the radiative decay rates. The black dashed curves show the energy of the guided mode without the exciton resonance. The horizontal gray dashed lines indicate the perovskite exciton energy.

To examine modes of the hybrid optical fiber, we use the well-known transfer matrix method, see Methods. Due to the presence of an evanescent field beyond the core of the waveguide, the polariton mode couples to the surface plasmon mode at the metal-core interface^30^. The characteristic guided modes of the hybrid waveguide are shown in Fig. 1(b). The dependence of the energy of the fundamental polariton mode on the propagation constant *β* in the hybrid optical fiber with the core diameter of 0.8 *μ*m is shown in Fig. 2(a). In the vicinity of the exciton resonance at *ω* ≈ *ω*_t_ and *β* ≈ 18.3 *μ*m^−1^ a clear Rabi splitting by $2\hslash {\Omega }_{{\rm{R}}}\approx 165\,{\rm{meV}}$ of the dispersion curve *ω*(*β*) into two branches is apparent, which results from the anti-crossing of the exciton and the guided optical mode dispersions. The dispersions of the latter being linear in *β* are shown in Fig. 2(a) by the dashed lines. The anti-crossing of the dispersions is the manifestation of the appearance of the coupled exciton-photon states, exciton polaritons, which we consider for the role of mediators of the superconductivity. An indispensable condition for the appearance of polaritons is the strong coupling condition, which implies that the characteristic losses in the system should not exceed the splitting $2\hslash {\Omega }_{{\rm{R}}}$. The color shadows framing the dispersion curves in Fig. 2(a) show the radiative broadening of the polariton modes. One can see that the Rabi spitting $2\hslash {\Omega }_{{\rm{R}}}$ exceeds the losses with a large margin.

Figure 2(b) shows the dependence of the exciton-polariton energy on the diameter of the core *d*_C_ for the polariton modes characterized by the wave number *β* = 18.3 *μ*m^−1^. The energy of polaritons of both branches increases with the decreasing diameter *d*_C_. The predominance of the Rabi splitting $2\hslash {\Omega }_{{\rm{R}}}$ over the characteristic losses on the entire considered range of *d*_C_ keeps the strong coupling condition fulfilled. By choosing the diameter of the glass core, one can tune characteristics of the guided polariton mode in the *ω* − *β* plane.

**Figure 3:**
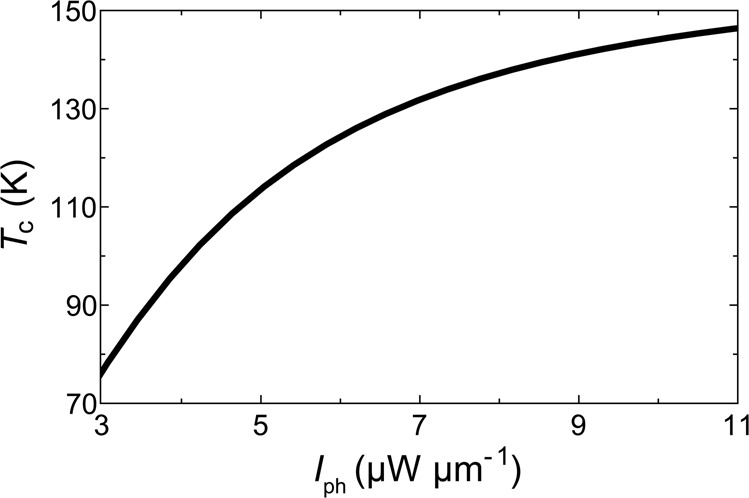
Critical temperature *T*_c_ of an aluminium superconductor being a cladding layer of the hybrid optical fiber as a function of the intensity of the photon field *I*_ph_.

To estimate the effect of coupling with the polariton mode on the critical temperature *T*_c_ of a metallic layer, we use the universal expression obtained in ref. ^14^:

2 $${k}_{{\rm{B}}}{T}_{{\rm{c}}}\approx 1.13\hslash {\omega }_{{\rm{B}}}\exp \left\{-,\frac{1}{{\eta }_{1}+{\eta }_{2}{[1-{\eta }_{2}{\rm{l}}{\rm{n}}({\omega }_{{\rm{D}}}/{\omega }_{{\rm{B}}})]}^{-1}}\right\},$$

where *η*_1_ and *η*_2_ are the effective coupling constants due to the excitations of the polariton condensate (bogolons) and due to phonons, respectively. *ω*_D_ and *ω*_B_ are the phonon and bogolon cut off frequencies. Figure 3 shows the increase of the critical temperature *T*_c_ of superconductivity in an aluminium layer with the increasing intensity of the light field in the waveguide for the parameters given in Methods. It is important to note that the scattering of electrons on a Fermi surface with exciton-polaritons contributes to the formation of Couper pairs also below the threshold to polariton lasing. However, the interaction of an electron with a thermal cloud of *N* excitons is expected to be somewhat weaker than its interaction with a coherent state of *N* excitons.

The fiber geometry with a perovskite coating layer has been proposed recently for fiber-shaped solar cells^31^. At the dawn of its appearance, this idea has faced difficulties in manufacturing since the conventional methods of fabricating planar structures including printing, spraying, spin-coating cannot be used for fibers. At the moment, various methods have been developed for covering a fiber surface with thin perovskite films^32–34^ which we expect are suitable for fabricating the proposed hybrid waveguides. In ref. ^32^ the dip-coating methods was used, which in contrast to spin-coating is not restricted by substrate size and shape. Both the single-step and the sequential-step dipping techniques we considered, which differ by a better coverage of perovskite on wire in the latter case. The authors of ref. ^34^ argue that the dip-coating method has the disadvantages poor crystal quality and low coverage. In turn, they suggest a vapor-assisted deposition method improved for the fiber geometry and providing a high-quality perovskite film layer on the fiber substrate. The metallic coating layer can be deposited, e.g. by a conventional methods of electro^35^ or electroless plating^36^. Along with the fabrication methods, the measurement techniques can be adopted from the fiber-shaped and planar solar cells. Among them are microscopy-based materials characterization, including the field emission scanning and transmission electron microscopy for the structure and morphology of the films, X-ray difraction measurements for characterizing a crystal structure of the perovskite cladding layer, surface and interface characterization, including X-ray photoelectron spectroscopy, Auger electron spectroscopy, characterization of optical properties, including photoemission spectroscopy, transient absorbance spectroscopy^33,34,37,38^.

In summary, we have proposed the structure of a hybrid optical fiber intended to provide an effective interaction of a superconductor with exciton polaritons, guided modes modified by coupling with excitons in a perovskite cladding layer. We have demonstrated that the strong-coupling regime for exciton polariton formation is realizable in this geometry. Exciton polaritons will fulfil the role of mediators of coupling of electrons in a superconductor which is expected to facilitate elevating the critical temperature of superconductivity.

## Methods

### Transfer matrix method for searching guided modes

To obtain the radial distribution of the electromagnetic field of a guided mode, we use a transfer matrix method^6,39–42^ adapted for a cylindrical geometry^43–45^. The electric and magnetic fields of a waveguide mode in a general case have the form

3 $${\bf{E}}({\bf{r}})={\bf{E}}(\rho )\exp [{\rm{i}}(\beta z+m\varphi )],\,{\bf{H}}({\bf{r}})={\bf{E}}(\rho )\exp [{\rm{i}}(\beta z+m\varphi )]$$

in the cylindrical coordinates **r** = (*ρ*, *φ*, *z*). The parameter $m\in {\mathbb{Z}}$ describes the azimuths variation of the fields. The transfer matrix method is based on matching the components of the electromagnetic field at the interfaces of the homogeneous layers using the Maxwell boundary conditions. The following equation

4 $${\bf{F}}(\rho )=\widehat{T}({\rho }_{0},\rho ){\bf{F}}({\rho }_{0})$$

for the vector **F**(*ρ*) = [*E*_*z*_(*ρ*), *H*_*ρ*_(*ρ*), *H*_*z*_(*ρ*), *E*_*ρ*_(*ρ*)]^T^ allows linking the field at a given radius *ρ* with one at another radius *ρ*_0_. $\widehat{T}({\rho }_{0},\rho )$ is the 4 × 4 transfer matrix through the homogeneous layer bound by radii *ρ*_0_ and *ρ*. In the special case of *m* = 0, one can separate the four-component vector **F**(*ρ*) into two-component vectors **F**_TM_(*ρ*) = [*E*_*z*_(*ρ*), *H*_*ρ*_(*ρ*)]^T^ and **F**_TE_(*ρ*) = [*H*_*z*_(*ρ*), *E*_*ρ*_(*ρ*)]^T^ for the TM- and TE-polarized modes, respectively. Equation (4) in this case splits into two equations

5 $${{\bf{F}}}_{{\rm{TM}},{\rm{TE}}}(\rho )={\widehat{T}}_{{\rm{TM}},{\rm{TE}}}({\rho }_{0},\rho ){{\bf{F}}}_{{\rm{TM}},{\rm{TE}}}({\rho }_{0})$$

with the 2 × 2 transfer matrices ${\widehat{T}}_{{\rm{TM}},{\rm{TE}}}({\rho }_{0},\rho )$ for the corresponding polarization components. The elements of the matrix ${\widehat{T}}_{{\rm{TM}}}({\rho }_{0},\rho )$ are as follows:

6a $$\begin{array}{ccc}{T}_{11} & = & \frac{\pi k{\rho }_{0}}{2}[{Y}_{0}^{{\rm{{\prime} }}}(k{\rho }_{0}){J}_{0}(k\rho )-{J}_{0}^{{\rm{{\prime} }}}(k{\rho }_{0}){Y}_{0}(k\rho )],\end{array}$$

6b $$\begin{array}{ccc}{T}_{12} & = & \frac{{\rm{i}}\pi {k}^{2}{\rho }_{0}}{2{k}_{0}{n}^{2}}[{Y}_{0}(k{\rho }_{0}){J}_{0}(k\rho )-{J}_{0}(k{\rho }_{0}){Y}_{0}(k\rho )],\end{array}$$

6c $$\begin{array}{ccc}{T}_{21} & = & \frac{{\rm{i}}\pi {n}^{2}{k}_{0}{\rho }_{0}}{2}[{Y}_{0}^{{\rm{{\prime} }}}(k{\rho }_{0}){J}_{0}^{{\rm{{\prime} }}}(k\rho )-{J}_{0}^{{\rm{{\prime} }}}(k{\rho }_{0}){Y}_{0}^{{\rm{{\prime} }}}(k\rho )],\end{array}$$

6d $$\begin{array}{ccc}{T}_{22} & = & \frac{\pi k{\rho }_{0}}{2}[{Y}_{0}^{{\rm{{\prime} }}}(k\rho ){J}_{0}(k{\rho }_{0})-{J}_{0}^{{\rm{{\prime} }}}(k\rho ){Y}_{0}(k{\rho }_{0})],\end{array}$$

where *J*_0_(*kρ*) and *Y*_0_(*kρ*) are zero order Bessel functions of the first and second kind, respectively. *k* is the radial component of the mode wave vector. We omit the subscript “*ρ*” for convenience. The elements of the transfer matrix ${\widehat{T}}_{{\rm{TE}}}({\rho }_{0},\rho )$ are obtained from Eq. (6) by applying the replacement *n*^2^ → *n*^*−*2^ to them. One could rewrite Eq. (6) in terms of Hankel functions ${H}_{0}^{(1)}(k\rho )$ and ${H}_{0}^{(2)}(k\rho )$ more suitable for describing problems with complex parameters. For the choice of a numerically satisfactory pair of solutions we refer to ref. ^46^. Based on the requirement to the field that it must be finite in the center of the waveguide (at *ρ* = 0), at the interface of the core and the perovskite cladding layer (at *ρ* = *ρ*_C_ ≡ *d*_C_/2) the vector **F**_TM_(*ρ*) takes the form

7 $${{\bf{F}}}_{{\rm{T}}{\rm{M}}}({\rho }_{{\rm{C}}})=\left(\begin{array}{c}{J}_{0}({k}_{{\rm{C}}}{\rho }_{{\rm{C}}})\\ \frac{i{k}_{0}{n}_{{\rm{C}}}^{2}}{{k}_{{\rm{C}}}}{J}_{0}^{{\rm{{\prime} }}}({k}_{{\rm{C}}}{\rho }_{{\rm{C}}})\end{array}\right){B}_{1}={\hat{t}}_{{\rm{C}}}{B}_{1},$$

where *k*_C_ is the radial wave vector component in the core, *B*_1_ is the constant. Outside the waveguide, the decaying solution of the guided mode at the interface of the superconducting cladding layer and air (at *ρ* = *ρ*_A_) is described by the vector

8 $${{\bf{F}}}_{{\rm{T}}{\rm{M}}}({\rho }_{{\rm{A}}})=\left(\begin{array}{c}{K}_{0}(|{k}_{{\rm{A}}}|{\rho }_{{\rm{A}}})\\ \frac{i{k}_{0}{n}_{{\rm{A}}}^{2}}{|{k}_{{\rm{A}}}|}{K}_{0}^{{\rm{{\prime} }}}(|{k}_{{\rm{A}}}|{\rho }_{{\rm{A}}})\end{array}\right){B}_{2}={\hat{t}}_{{\rm{A}}}{B}_{2},$$

where *n*_A_ = 1 and *k*_A_ are the refractive index and the radial wave vector component in air, *B*_2_ is a constant, *K*_0_(*kρ*) is the zero order modified Bessel function.

At the interface of the superconducting layer and air, the electromagnetic field obeys the equation ${\widehat{T}}_{{\rm{TM}}}{{\bf{F}}}_{{\rm{TM}}}({\rho }_{{\rm{C}}})={{\bf{F}}}_{{\rm{TM}}}({\rho }_{{\rm{A}}})$ which can be transformed to

9 $$({\hat{T}}_{{\rm{T}}{\rm{M}}}{\hat{t}}_{{\rm{C}}}|-\,{\hat{t}}_{{\rm{A}}})(\begin{array}{c}{B}_{1}\\ {B}_{2}\end{array})=0,$$

where ${\widehat{T}}_{{\rm{TM}}}$ is the appropriate transfer matrix through the layers between the radii *ρ*_C_ and *ρ*_A_. The energies of the guided modes obey the condition: the determinant of the 2 × 2 matrix $({\widehat{T}}_{{\rm{TM}}}{\hat{t}}_{{\rm{C}}}|\,-\,{\hat{t}}_{{\rm{A}}})$ is equal to zero. For the TE modes, the eigenmode condition can be found in a similar way. In the case of the nonzero azimuth variation, *m* ≠ 0, the TE and TM modes can’t be uncoupled, and a similar procedure should be applied directly to the four-component vector **F**(*ρ*).

### Estimation of the parameters used for calculations

To estimate the critical temperature of aluminium in the hybrid waveguide, we take the following values of the parameters. The aluminium layer considered as a superconducting layer following ref. ^14^ is taken to be characterized by the effective electron-phonon coupling constant *η*_2_ = 0.3. The phonon cut off frequency approximated by the Debye frequency is $\hslash {\omega }_{{\rm{D}}}/{k}_{{\rm{B}}}=429\,{\rm{K}}$. The effective electron-bogolon coupling constant *η*_1_ is estimated for the given configuration as following:

10 $${\eta }_{1}=\frac{{X}^{4}{L}^{2}{d}^{2}{e}^{2}{n}_{p}^{2}AN(0)}{{g}_{0}{\varepsilon }^{2}},$$

where *X* is the Hopfield coefficient which determines the exciton fraction in the polariton state. *L* is the distance between the metallic layer and the perovskite layer, *d* is a dipole momentum of excitons, *n*_p_ is the polariton number density in the guided mode. *A* is the normalization area, *N*(0) is the electron density of states for electrons, characterized by the effective mass *m*_e_, *g*_0_ is the two-particle polariton interaction constant. *e* and *ε* are the elementary charge and the absolute permittivit, respectively.

The exciton fraction *X* is a controlable parameter which can be set in a range of [0, 1] by choosing an appropriate detuning of the waveguide light mode frequency and the perovskite exciton frequency. For our estimations, we take $X=1/\sqrt{2}$ corresponding to the parity of the contributions of the light and exciton components. The separation of the electron gas and the polariton condensate is estimated as the distance between the centers of the metallic and perrovskite layers and is taken as *L* = 25 nm. Excitons in the perovskite layer possess a non-zero dipole moment *d* which can be either built-in due to an internal piezoelectric field or induced by an externally applied electric field^8^. For our estimations, we take *d*/*e* = 10 nm. The exciton-exciton interaction constant in the perovskite was estimated in ref. ^47^ as 3 *μ*eV*μ*m^2^, which exceeds the polariton-polariton interaction constant by the factor of *X*^−4^. For our estimations, we take the interaction energy as *g*_0_/*A* = 0.01 *μ*eV. The bogolon cut off frequency is taken for our estimations as $\hslash {\omega }_{{\rm{B}}}/{k}_{{\rm{B}}}=140\,{\rm{K}}$.

The intensity of the light field along the waveguide is estimated as follows: ${I}_{{\rm{ph}}}\simeq 2\pi \hslash {D}_{{\rm{P}}}{\omega }_{{\rm{ph}}}{\gamma }_{{\rm{ph}}}{C}^{2}{n}_{{\rm{p}}}$, where $\hslash {\omega }_{{\rm{ph}}}$ and 2*γ*_ph_ are the energy of the resonant optical mode in the waveguide and the light intensity decay rate, respectively. The optical mode which contributes to the polariton state considered in Fig. 2 and propagating with the wave number *β* = 18.3 *μ*m^−1^ is characterized by $\hslash {\omega }_{{\rm{ph}}}=2.303$ eV and $2{\gamma }_{{\rm{ph}}}\simeq 79\,{{\rm{ps}}}^{-1}$. *C*^2^ determines the light fraction in the polariton state, *C*^2^ = 1 − *X*^2^. Based on the fact that the considered ground guided polariton state is azimuthally symmetric, we introduce the 1D polariton density along the waveguide as *πD*_P_*n*_P_, where *D*_P_ is the diameter of the interface of the perovskite layer and the metallic layer.

## References

[1] Bardeen J, Cooper LN, Schrieffer JR (1957). Microscopic theory of superconductivity. Phys. Rev..

[2] Allender D, Bray J, Bardeen J (1973). Model for an exciton mechanism of superconductivity. Phys. Rev. B.

[3] Ginzburg, V. L. High-temperature superconductivity – dream or reality? *Usp. Fiz. Nauk*. **118**, 315, 10.3367/UFNr.0118.197602f.0315 (1976) [Sov. Phys. Usp. **19**, 174, 10.1070/PU1976v019n02ABEH005136 (1976)].

[4] Kasprzak J (2006). Bose–Einstein condensation of exciton polaritons. Nature.

[5] Christopoulos S (2007). Room-temperature polariton lasing in semiconductor microcavities. Phys. Rev. Lett..

[6] Kavokin, A. V., Baumberg, J. J., Malpuech, G. & Laussy, F. P. (eds.) *Microcavities*, 2 edn (Oxford University Press, Oxford, 2017).

[7] Laussy FP, Kavokin AV, Shelykh IA (2010). Exciton-polariton mediated superconductivity. Phys. Rev. Lett..

[8] Laussy FP, Taylor T, Shelykh IA, Kavokin AV (2012). Superconductivity with excitons and polaritons: review and extension. J. Nanophotonics.

[9] Cherotchenko E, Espinosa-Ortega T, Nalitov A, Shelykh I, Kavokin A (2016). Superconductivity in semiconductor structures: The excitonic mechanism. Superlattices Microstruct..

[10] Cotle¸t O, Zeytinoğlu S, Sigrist M, Demler E, Imamoğlu A (2016). Superconductivity and other collective phenomena in a hybrid Bose-Fermi mixture formed by a polariton condensate and an electron system in two dimensions. Phys. Rev. B.

[11] Kavokin A, Lagoudakis P (2016). Exciton-mediated superconductivity. Nat. Mater..

[12] Myers DM (2018). Superlinear increase of photocurrent due to stimulated scattering into a polariton condensate. Phys. Rev. B.

[13] Su R (2017). Room-temperature polariton lasing in all-inorganic perovskite nanoplatelets. Nano Lett..

[14] Skopelitis P, Cherotchenko ED, Kavokin AV, Posazhennikova A (2018). Interplay of phonon and excitonmediated superconductivity in hybrid semiconductor-superconductor structures. Phys. Rev. Lett..

[15] Fausti, D. *et al*. Light-induced superconductivity in a stripe-ordered cuprate. *Science* **331**, 189–191, 10.1126/science.1197294https://science.sciencemag.org/content/331/6014/189.full.pdf (2011). 10.1126/science.1197294 21233381

[16] Mitrano, M. *et al*. Possible light-induced superconductivity in K_3_C_60_ at high temperature. *Nature* **530**, 461–464, 10.1038/nature16522 (2016). 10.1038/nature16522 PMC4820655 26855424

[17] Tong, L., Zi, F., Guo, X. & Lou, J. Optical microfibers and nanofibers: A tutorial. *Opt. Commun*. **285**, 4641**–**4647, 10.1016/j.optcom.2012.07.068 Special Issue: Optical micro/nanofibers: Challenges and Opportunities. (2012).

[18] Zhang S (2018). Strong exciton–photon coupling in hybrid inorganic–organic perovskite micro/nanowires. Adv. Opt.Mater..

[19] Zhu H (2015). Lead halide perovskite nanowire lasers with low lasing thresholds and high quality factors. Nat. Mater..

[20] Khukhareva, I. S. The superconducting properties of thin aluminium films. *J. Exptl. Theoret. Phys*. **43**, 1173–1178 (1962) [Sov. Phys. JETP 16, 828–832 (1963)].

[21] Carusotto I, Ciuti C (2013). Quantum fluids of light. Rev. Mod. Phys..

[22] Zhang Q (2017). Advances in small perovskite-based lasers. Small Methods.

[23] Leguy AMA (2016). Experimental and theoretical optical properties of methylammonium lead halide perovskites. Nanoscale.

[24] Kato M (2017). Universal rules for visible-light absorption in hybrid perovskite materials. J. Appl. Phys..

[25] McPeak KM (2015). Plasmonic films can easily be better: Rules and recipes. ACS Photonics.

[26] Su, R. *et al*. Room temperature long-range coherent exciton polariton condensate flow in lead halide perovskites. *Sci. Adv*. **4**, 10.1126/sciadv.aau0244https://advances.sciencemag.org/content/4/10/eaau0244.full.pdf (2018). 10.1126/sciadv.aau0244 PMC6203223 30397645

[27] Laming, R. I. *et al*. Erbium Doped Fibre Lasers And Amplifiers. In Digonnet, M. J. F. (ed.) *Fiber Laser Sources and Amplifiers*, vol. 1171, 82–92, 10.1117/12.963141. International Society for Optics and Photonics (SPIE, 1990).

[28] Zervas MN, Laming RI, Townsend JE, Payne DN (1992). Design and fabrication of high gain-efficiency erbium-doped fiber amplifiers. IEEE Photonics Technol. Lett..

[29] Laming RI, Zervas MN, Payne DN (1992). Erbium-doped fiber amplifier with 54 dB gain and 3.1 dB noise figures. IEEE Photonics Technol. Lett..

[30] Nasirifar R, Danaie M, Dideban A (2019). Dual channel optical fiber refractive index sensor based on surface plasmon resonance. Optik.

[31] Qiu L, Deng J, Lu X, Yang Z, Peng H (2014). Integrating perovskite solar cells into a flexible fiber. Angewandte Chemie Int. Ed..

[32] Wang X (2016). Wire-shaped perovskite solar cell based on TiO_2_ nanotubes. Nanotechnology.

[33] Hu H, Dong B, Chen B, Gao X, Zou D (2018). High performance fiber-shaped perovskite solar cells based on lead acetate precursor. Sustain. Energy Fuels.

[34] Dong B (2019). High-efficiency fiber-shaped perovskite solar cell by vapor-assisted deposition with a record efficiency of 10.79%. Adv. Mater. Technol..

[35] Sandlin S, Kinnunen T, Rämö J, Sillanpää M (2006). A simple method for metal re-coating of optical fibre Bragg gratings. Surf. Coatings Technol..

[36] Bialiayeu A, Caucheteur C, Ahamad N, Ianoul A, Albert J (2011). Self-optimized metal coatings for fiber plasmonics by electroless deposition. Opt. Express.

[37] Aguiar JA (2016). Effect of water vapor, temperature, and rapid annealing on formamidinium lead triiodide perovskite crystallization. ACS Energy Lett..

[38] Schulz P (2016). Charge transfer dynamics between carbon nanotubes and hybrid organic metal halide perovskite films. The J. Phys. Chem. Lett..

[39] Chew,W. C. *Waves and Fields in Inhomogeneous Media* (Wiley-IEEE Press, New York, 1995).

[40] Sedov ES, Cherotchenko ED, Arakelian SM, Kavokin AV (2016). Light propagation in tunable exciton-polariton one-dimensional photonic crystals. Phys. Rev. B.

[41] Sedov, E. S., Iorsh, I. V., Arakelian, S. M., Alodjants, A. P. & Kavokin, A. Hyperbolic Metamaterials with Bragg Polaritons. *Phys. Rev. Lett.* **114**, 237402, 10.1103/PhysRevLett.114.237402 (2015). 10.1103/PhysRevLett.114.237402 26196825

[42] Sedov, E. S. *et al*. Hyperbolic metamaterials based on Bragg polariton structures. Pis’ma ZhETF **104**, 58-63 (2016) [JETP Letters **104**, 62–67, 10.1134/S0021364016130130 (2016)].

[43] Kaliteevskii, M. A., Nikolaev, V. V. & Abram, R. A. Calculation of the mode structure of multilayer optical fibers based on transfer matrices for cylindrical waves. *Optika i Spektroskopiya* **88**, 871 (2000) [Optics and Spectroscopy 88, 792 (2000)].

[44] Little CE (2012). Tamm plasmon polaritons in multilayered cylindrical structures. Phys. Rev. B.

[45] Kaliteevski MA (2000). Exciton polaritons in a cylindrical microcavity with an embedded quantum wire. Phys. Rev. B.

[46] Temme, N. M. *Special Functions: An Introduction to the Classical Functions of Mathematical Physics* (John Wiley & Sons, New York, 1996).

[47] Fieramosca, A. *et al*. Two-dimensional hybrid perovskites sustaining strong polariton interactions at room temperature. *Sci. Adv*. **5**, 10.1126/sciadv.aav9967https://advances.sciencemag.org/content/5/5/eaav9967.full.pdf (2019). 10.1126/sciadv.aav9967 PMC6544457 31172027

